# Electrowetting lens with large aperture and focal length tunability

**DOI:** 10.1038/s41598-020-73260-4

**Published:** 2020-10-01

**Authors:** Xiaomin Song, Hongxia Zhang, Dongyang Li, Dagong Jia, Tiegen Liu

**Affiliations:** 1 College of Precision Instrument and Optoelectronics Engineering, Tianjin University, Tianjin, 300072 China; 2 Key Laboratory of Optoelectronics Information Technical Science, EMC, Tianjin, 300072 China

**Keywords:** Fluid dynamics, Adaptive optics, Optoelectronic devices and components

## Abstract

The electrowetting lenses has attracted researchers in many fields, such as biology, beam shaping, and drug delivery. Previous research on electrowetting lens has focused on neither expanding the dynamic focal length range nor reducing the wavefront aberration. However, the properties with large numerical aperture and low aberration are also essential properties of lenses, and can promote their application. Therefore, we calculated the meniscus of the lens with different optical apertures, and subsequently, analyzed the relations among the focal length, wavefront aberration, and optical aperture. To expand the focal length range, we designed an electrowetting-based triple-liquid lens with a root-mean-square wavefront aberration error of less than 1/4 waves.

## Introduction

Electrowetting lenses are widely applied in imaging 3D biological activities ^1–3^, beam shaping ^4^, correcting wavefront aberration ^5^, display ^6^, and drug delivery ^7^. One of their significant characteristics is focal length tunability, which results from the liquid–liquid interface generated by an additional electric field ^8^. The main parameters of an adaptive liquid lens include optical aperture, wavefront aberration, and dynamic focal length range. However, most previous studies have focused on neither optical aperture nor wavefront aberration. For example, Wang et al. designed and implemented a variable-focus lens with a large optical aperture, based on the liquid–membrane–liquid structure ^9^. Lee et al. designed and implemented a micro-electro-mechanical system-based tunable liquid micro-lens with an aperture of 30 mm, which exhibited a tuning range of back focal length between 2.3 mm and infinity ^10^. Zohrabi et al. presented numerical simulations of multielectrode electrowetting devices used in a novel optical design for obtaining correct wavefront aberration ^11^. Kim et al. designed an electrowetting lenticular lens with high diopter, using a lens-shaped ETPTA(trimethylopropane ethoxylate triacrylate) chamber ^12^. Kopp et al. proposed an all dual-lens optofluidic zoom system whose focal length could be expanded using the two combined liquid lens method ^13^. Wang et al. proposed a hybrid driving variable-focus optofluidic lens, which had a large zoom range and high ability for obtaining correct aberrations ^14^.

As the numerical aperture, which depends on the optical aperture and focal length, is also a vital property of a lens, determines the spatial resolution, in this study, we analyze the focal length range, optical aperture, and wavefront aberration value under different applied voltages; compare the resolution of the lens with the same focal length. The study results indicate that the focal length range is directly proportional to the optical aperture and the wavefront aberration is directly proportional to the absolute value of the optical power. According to the above characteristics, we propose an electrowetting-based triple-liquid lens whose numerical aperture is larger than those of traditional electrowetting lens.

## Geometrical configuration of liquid–liquid interface

**Figure 1:**
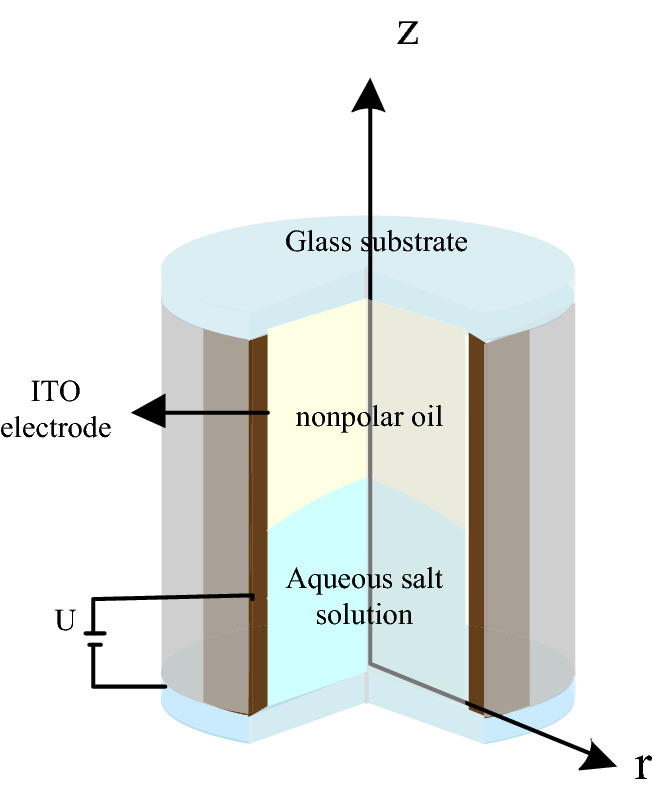
Schematic view of EWOD lens.

In this study, an electrowetting lens configuration was completely fabricated on a cylindrical coordinate system (*r, z*) in COMSOL Multiphysics, which was normalized with reference length L (a half-lens aperture). As illustrated in Fig. 1, the geometric configuration simulation in COMSOL Multiphysics was used in the actual lens ^15^. The lens device was constructed in a cylindrical housing with an indium-tin-oxide sidewall. We applied Telfon (~ 500 nm) as the hydrophobic layer and Parylene-N (~ 3 μm) as the dielectric layer. We used two immiscible liquids with different refractive indices, where an aqueous salt solution, used as a conductive liquid, was dropped on the bottom of the cylindrical glass tube and the nonpolar oil, used as an insulating liquid, was filled in the glass.

The curvature of the meniscus between two liquids can be expressed using the Young–Laplace equation ^14^ as follows:

1 $$k = Boh - Ca_{c} \left( {\overset{\lower0.5em\hbox{$\smash{\scriptscriptstyle\frown}$}}{p}_{l} - \overset{\lower0.5em\hbox{$\smash{\scriptscriptstyle\frown}$}}{p}_{s} } \right)$$

2 $$k = k_{1} + k_{2} ,\quad k_{1} = \frac{{h^{\prime\prime}}}{{\left( {1 + h^{{\prime}{2}} } \right)^{1.5} }},\quad k_{2} = \frac{{h^{\prime}}}{{r\left( {1 + h^{^{\prime}2} } \right)^{0.5} }}$$

3 $$Bo = \frac{{\left( {\rho_{l} - \rho_{s} } \right)gL^{2} }}{\gamma }$$

4 $$Ca_{c} = \frac{{\mu U_{c} }}{\gamma }$$

where $$z = h\left( r \right)$$ represents the meniscus profile; $$\mu$$ and $$\rho$$ represent viscosity and density, respectively; and the subscripts $$l$$ and $$s$$ denote aqueous salt and nonpolar oil solutions. The bond and capillary numbers are based on the reference velocity, $$U_{c}$$, and the primes $$\overset{\lower0.5em\hbox{$\smash{\scriptscriptstyle\frown}$}}{p}_{l}$$ and $$\overset{\lower0.5em\hbox{$\smash{\scriptscriptstyle\frown}$}}{p}_{s}$$, which denote the flow-induced pressure on the aqueous salt and nonpolar oil solutions, respectively. In an equilibrium state, the curvature $$k\left( r \right)$$ reduces to

5 $$k\left( r \right) = Bo\left( {h\left( r \right) - h\left( 0 \right)} \right) + k\left( 0 \right)$$

The primes represent *Bo* derivatives with respect to *r*, the capillary number *Cac* is based on $U_{c}$, and γ denotes the surface tension.

**Figure 2:**
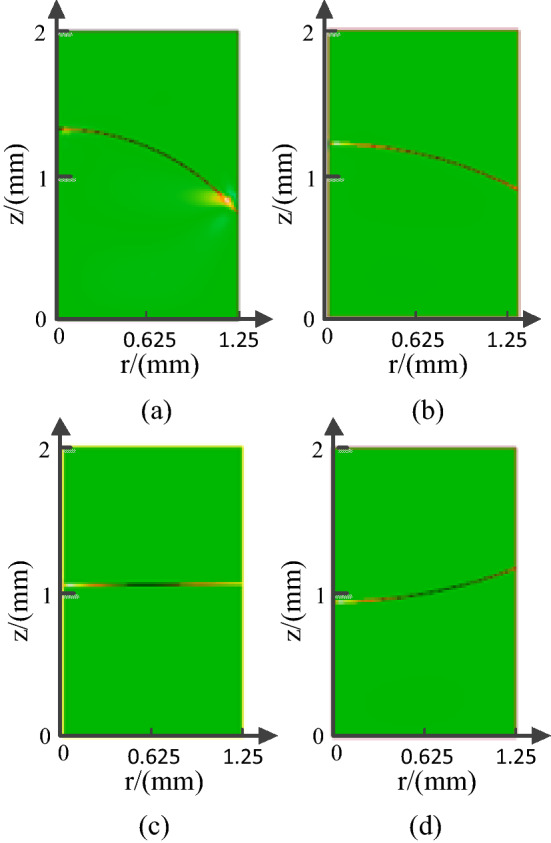
Meniscus of the EWOD lens in equilibrium state; from (**a**–**d**), the optical aperture D = 2.5 mm and the applied voltage *U* = 0 V, *U* = 60 V, *U* = 100 V and *U* = 120 V, respectively.

Equation (5) is a nonlinear ordinary differential equation for $h\left( r \right)$, whose associated boundary conditions are expressed as

6 $$h^{\prime}\left( 0 \right) = 0,\quad h^{\prime}\left( {r_{0} } \right) = \cot \theta$$

where $r$ denotes the surface of the cylinder hole. We can calculate the contact angle from the Lippmann equation as follows:

7 $$\cos \theta = \cos \theta_{0} + \frac{{\varepsilon_{d} \varepsilon_{0} }}{2\gamma d}U^{2}$$

where $d$ and $\varepsilon_{d}$ are the thickness and relative permittivity of the dielectric layer, respectively, $\theta_{0}$ is the contact angle in the absence of an electric field, and $\varepsilon_{0}$ is the vacuum permittivity $\left( {\varepsilon_{0} = 8.854 \times 10^{ - 12} } \right)$. Then, we can calculate the meniscus between the two immiscible liquids. Figure 2a shows the meniscus (liquid–liquid surface) of an electrowetting-on-dielectric (EWOD) lens with a 2.5 mm optical aperture in the absence of an electric field. Figures 2a–c show the meniscus profile under the induced voltages of 60, 100, and 120 V, respectively.

The meniscus profile depends on the contact angle, the original contact angle is 140°, and the meniscus is concave. It gradually decreases as the applied voltage increases, which reduces to the meniscus ranging from concave to convex. The contact angle tends to saturate at *U* = 120 V. According to Eq. (6), the optical aperture is another factor influencing the formed meniscus. Therefore, we studied the relation between the radius of the meniscus and optical aperture.

**Figure 3:**
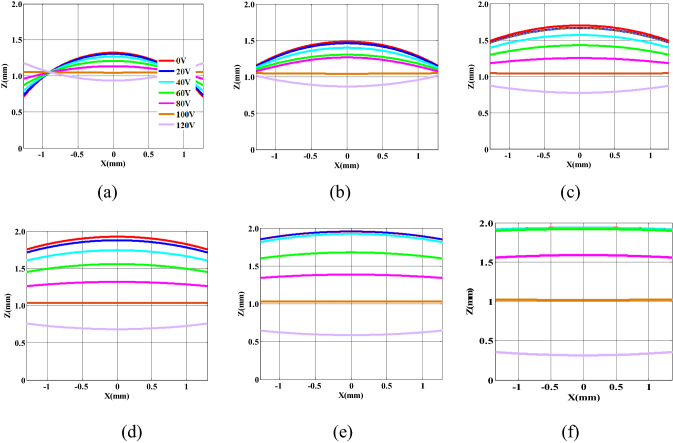
Meniscus of the EWOD lens with different optical apertures at various voltages: (**a**) 2.5 mm (**b**) 4 mm (**c**) 6 mm (**d**) 8 mm (**e**) 10 mm (**f**) 16 mm.

Because of the symmetry of the cylindrical tube, we can characterize the meniscus by a curve formed by its projection on the X–Z plane. To explore the relations among the radius of the meniscus, optical aperture, and applied voltage, we fitted the curve of the meniscus using the least square method, as shown in Fig. 3. The figure shows that as the applied voltage increases, the center height of the meniscus decreases, while its edge height increases. Because of the unfixed curvature of the meniscus, the electrowetting lens has a dynamic focal length range under the applied voltage.

**Figure 4:**
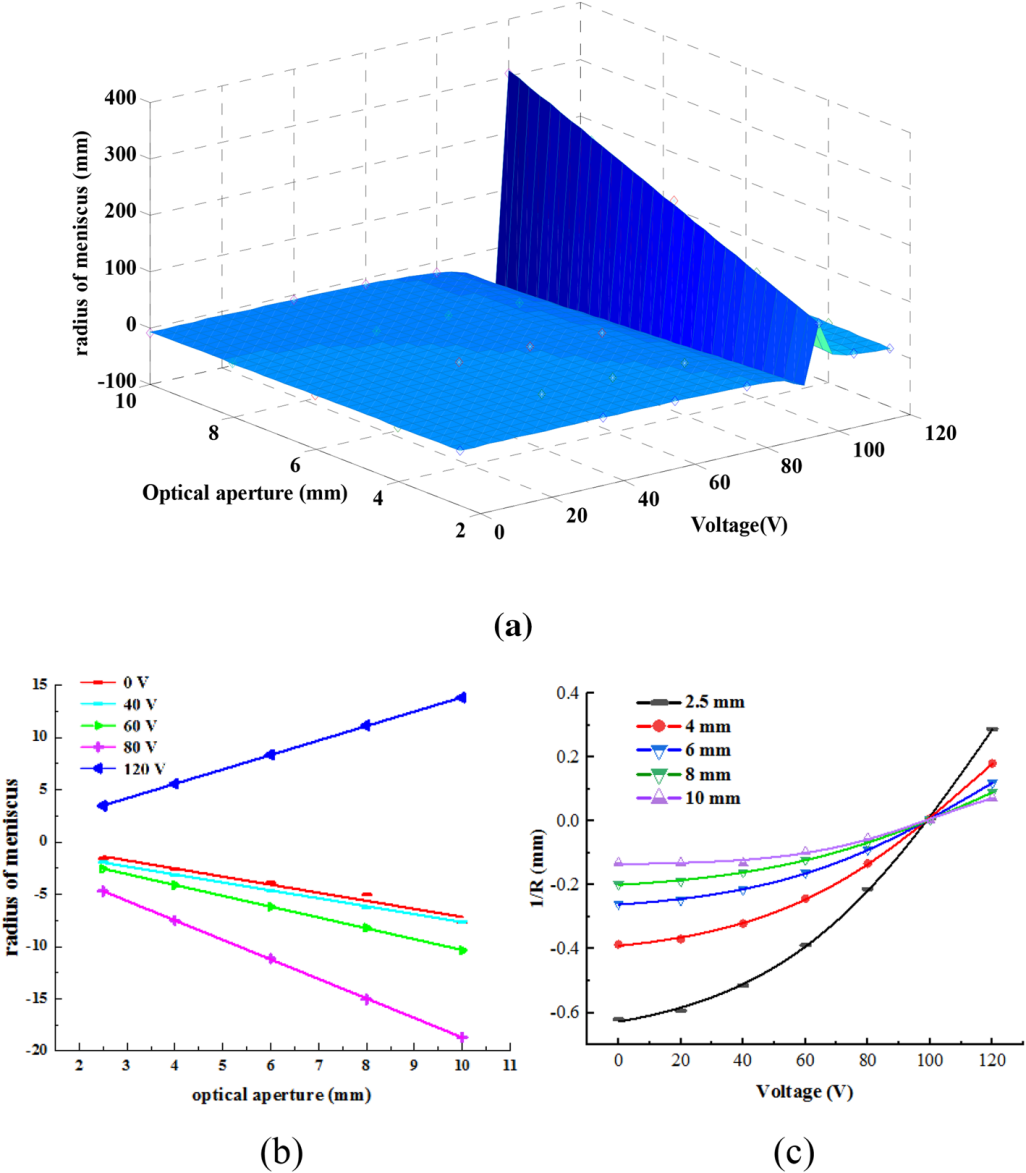
(**a**) Dependence of radius of meniscus on the optical aperture and applied voltage and the applied voltage, (**b**) the radius of meniscus is linearly related to the optical aperture, the slope of the curve has the value of $1/\cos \theta$, (**c**) the reciprocal of the radius of meniscus is proportional to the square of the applied voltage.

As shown in Fig. 2, we can configure the liquid lens to work in three schemes—converging, not deflecting, and diverging lights. Because the refractive index of the salt solution is smaller than that of the nonpolar oil solution, the lens exhibits the characteristics of a concave lens at *U* = 0 V, and a negative radius of meniscus is obtained, which decreases as the applied voltage increases. When *U* > 95 V, the lens exhibits the characteristics of a convex lens, and a positive radius of meniscus is obtained, which also decreases as the applied voltage increases. Comparing the meniscus of the lens with various optical apertures, we found that the radius of meniscus is directly proportional to the optical aperture. However, because of the low volume–area ratio, we could not observe the liquid–liquid surface as a lens, as shown in Fig. 3f, and thus, will not discuss the relation between either the optical aperture or the contact angle and the radius of meniscus here. Because the applied voltage, as a unique variable factor, decides the contact angle values, the relations among optical aperture, applied voltage, and radius of meniscus are as shown in Fig. 4.

As shown in Fig. 4, the radius of meniscus changes approximately linearly with the optical aperture, the coefficient depends on the contact angle if it is larger than $\pi /2$, and the coefficient is negative and decreases with the applied voltage; otherwise, the coefficient is positive and increases with the applied voltage. Furthermore, we inferred a quadratic equation of the reciprocal of the radius of meniscus and applied voltage. Because the meniscus is restructured with the spherical wave, we can express the radius of mensicus as follows:

8 $$R = \frac{r}{\cos \theta } = \frac{r}{{\cos \theta_{0} + \frac{\varepsilon }{{2\gamma_{12} d}}V^{2} }}$$

### Properties of the liquid lens

Focal length is one of the main properties of a liquid lens. Similar to the solid lens, the focal length of the liquid lens is also based on the radius of meniscus *R*, the refractive index of the two liquids $n_{w}$ and $n_{g}$, and the contact angle $\theta$.

9 $$f = \frac{r}{{n_{w} - n_{g} }}\frac{1}{{\cos \theta_{0} + \frac{\varepsilon }{{2\gamma_{12} d}}U^{2} }}$$

where $n_{w} ,n_{g}$ are the refractive indices of the conductive and insulating liquids, respectively.

**Figure 5:**
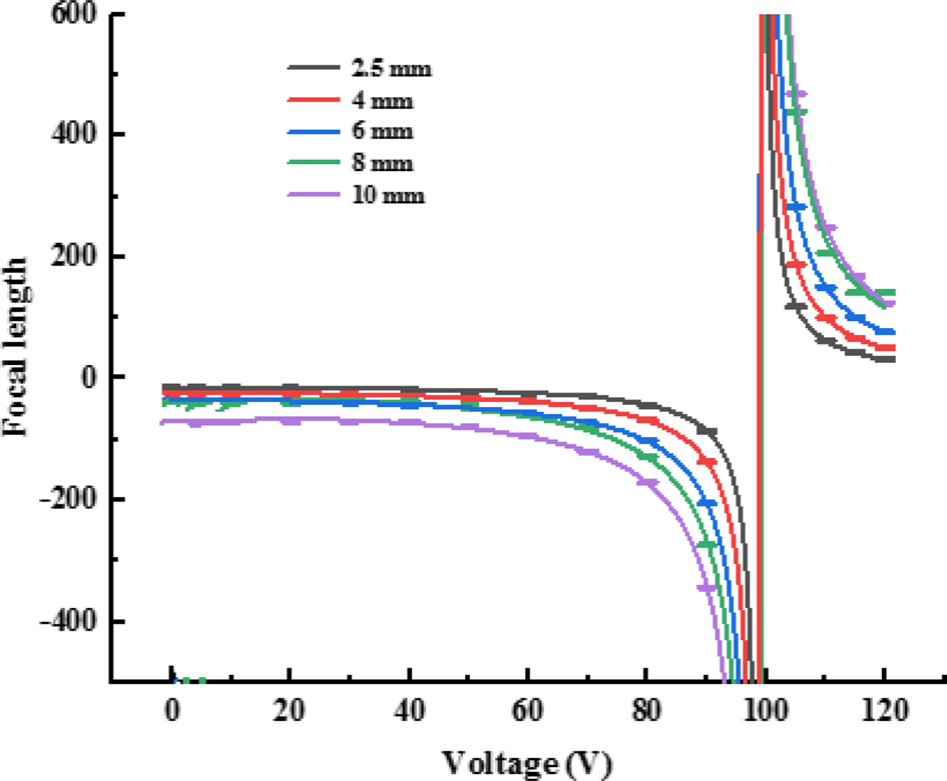
Dependence of the focal length of EWOD on the applied voltage and optical aperture.

To explore the focusing property, we imported the liquid lens structure into the raying trace Zemax. Figure 5 shows the predicted focal length of the EWOD lens of different optical aperture at various voltages.

Figure 5 reveals that the negative focal length decreases as the applied voltage increases, while it becomes positive when *V* = 100 V; then, it increases with the applied voltage. The focal length of the EWOD lens with 2.5 mm optical aperture can be tuned in the range (− ∞, − 14.658 mm) ∪ (31.5 mm, + ∞), the focal length range decreases as the optical aperture increases; when the radius of the cylindrical tube increases to 5 mm, we can only tune the focal length in the range (− ∞, − 73.260 mm) ∪ (77. 200 mm, + ∞).

According to the relationship between wavefront aberration and spherical aberration, the wavefront aberration depends on the square of the focal length and maximum incident height.

**Figure 6:**
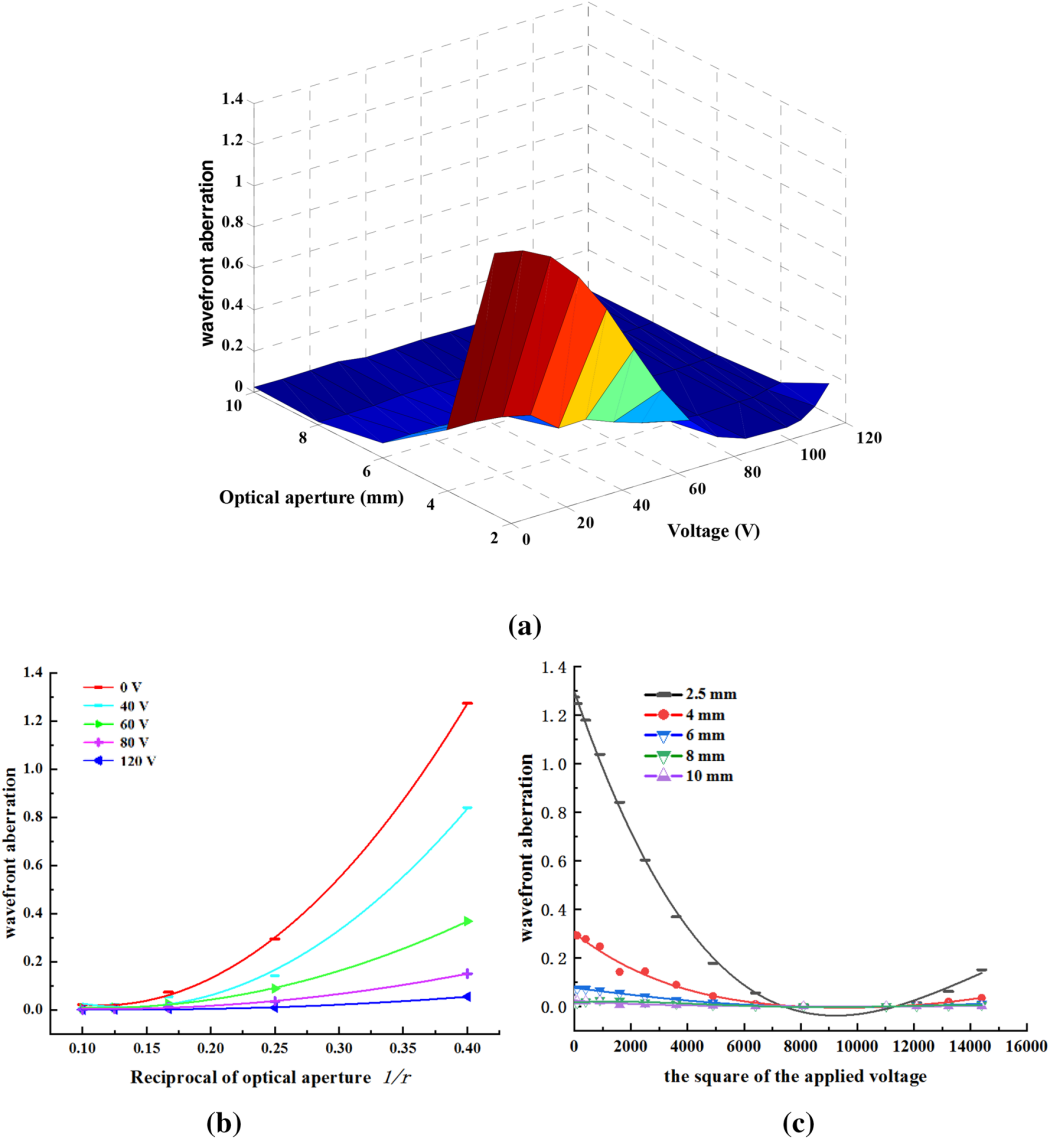
(**a**) Dependence of wavefront aberration on the optical aperture and applied voltage, (**b**) relation between wavefront aberration and optical aperture, (**c**) relation between wavefront aberration and applied voltage.

Here, we calculated the wavefront aberration to represent the quality of the EWOD lens, as shown in Fig. 6.

As shown in Fig. 6, the wavefront aberration depends on the optical aperture and applied voltage, which decreases as the optical aperture increases. Because the reciprocal of the focal length is directly proportional to the square of the applied voltage, the wavefront aberration is directly proportional to the reciprocal of the focal length.

We fitted the curve by using the regression analysis method, which revealed that $w \propto a_{0} \frac{1}{r} + a_{1} \frac{1}{{r^{2} }}$ and $w \propto b_{0} + b_{1} U^{2} + b_{2} U^{4} + b_{3} U^{6} + b_{4} U^{8}$ ($w$ is also a function of the square of the applied voltage), simultaneously.where $a_{0} ,a_{1} ,b_{0} ,b_{1} ,b_{2} ,b_{3} ,b_{4}$ are the fitting coefficients.

**Figure 7:**
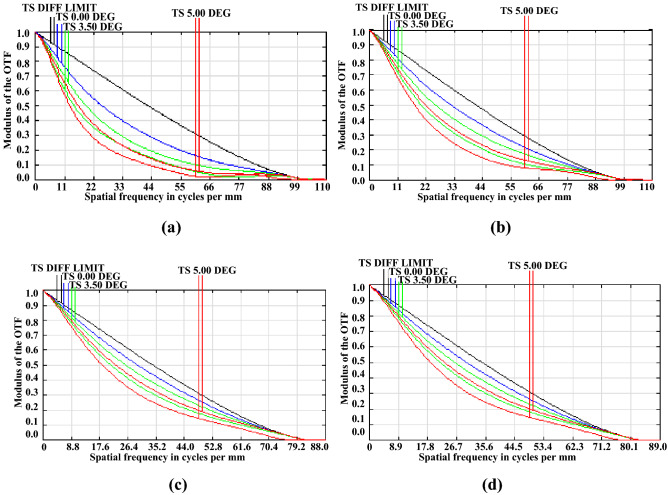
MTF curve of the EWOD lens: (**a**) D = 2.5 mm, f = -35.6 mm (**b**) D = 6 mm, f = − 35.6 mm (**c**) D = 2.5 mm, f = − 42. 5 mm, and (**d**) D = 6 mm, f = − 42. 5 mm, where D denotes the optical aperture and (**f**) denotes the focal length.

Numerical aperture, as a main parameter of a lens, determines the spatial resolution value, which is related to the focal length and optical aperture; therefore, we calculated the modulation transfer function (MTF) for a lens with similar focal length, as shown in Fig. 7. Figure 7a,b show an MTF curve where the lens’ optical aperture is 2.5 mm and 6 mm, respectively, while the focal length is − 35.6 mm; the focal length of the lens shown in Fig. 7c,d is − 42.5 mm.

As shown in Fig. 7, the parallel light passing through the adaptive lens is focused at different positions under the applied electric field, namely − 35.6 and − 42.6 mm; however, because of the lower aberration of the lens with large optical aperture, the spatial resolution of the lens with 2.5 mm optical aperture is lower than that of the lens with 6 mm optical aperture.

### Electrowetting-based triple-liquid dual-zoom lens

To expand the focal length of the EWOD lens under the condition of uniform wavefront aberration, we proposed an electrowetting-based triple-liquid dual-zoom lens that comprises three liquids—two conductive liquids and one insulative liquid. The double lens, which can be independently tuned, the compound mode with two convex lenses can improve the focusing quality, but the mode with a convex lens and a concave lens can expand the focus tuning range. In addition, the high tuning precision can be obtained by applying a voltage to the double liquid lens, respectively. Because of the limitation of the threshold voltage, the optical aperture with 6 mm has been chosen.

As shown in Fig. 3, the threshold voltage is 10 V, 10 V, 20 V, 40 V, 60 V at D = 2.5 mm, D = 6 mm, D = 8 mm, D = 10 mm, D = 16 mm, respectively(D is the optical aperture). In order to reduce the threshold voltage, improve the focusing quality, the optical aperture with 6 mm is chosen.

**Figure 8:**
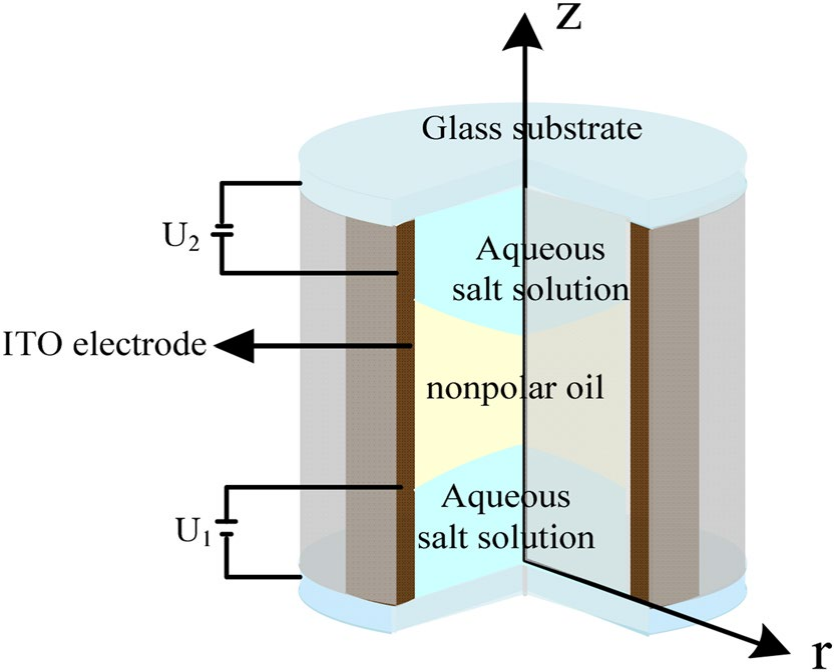
Crow view of the electrowetting-based triple-liquid dual-zoom lens.

We sandwiched the insulative liquid with the two conductive ones, as shown in Fig. 8. In this study, both conductive liquids were aqueous salt solutions.

**Figure 9:**
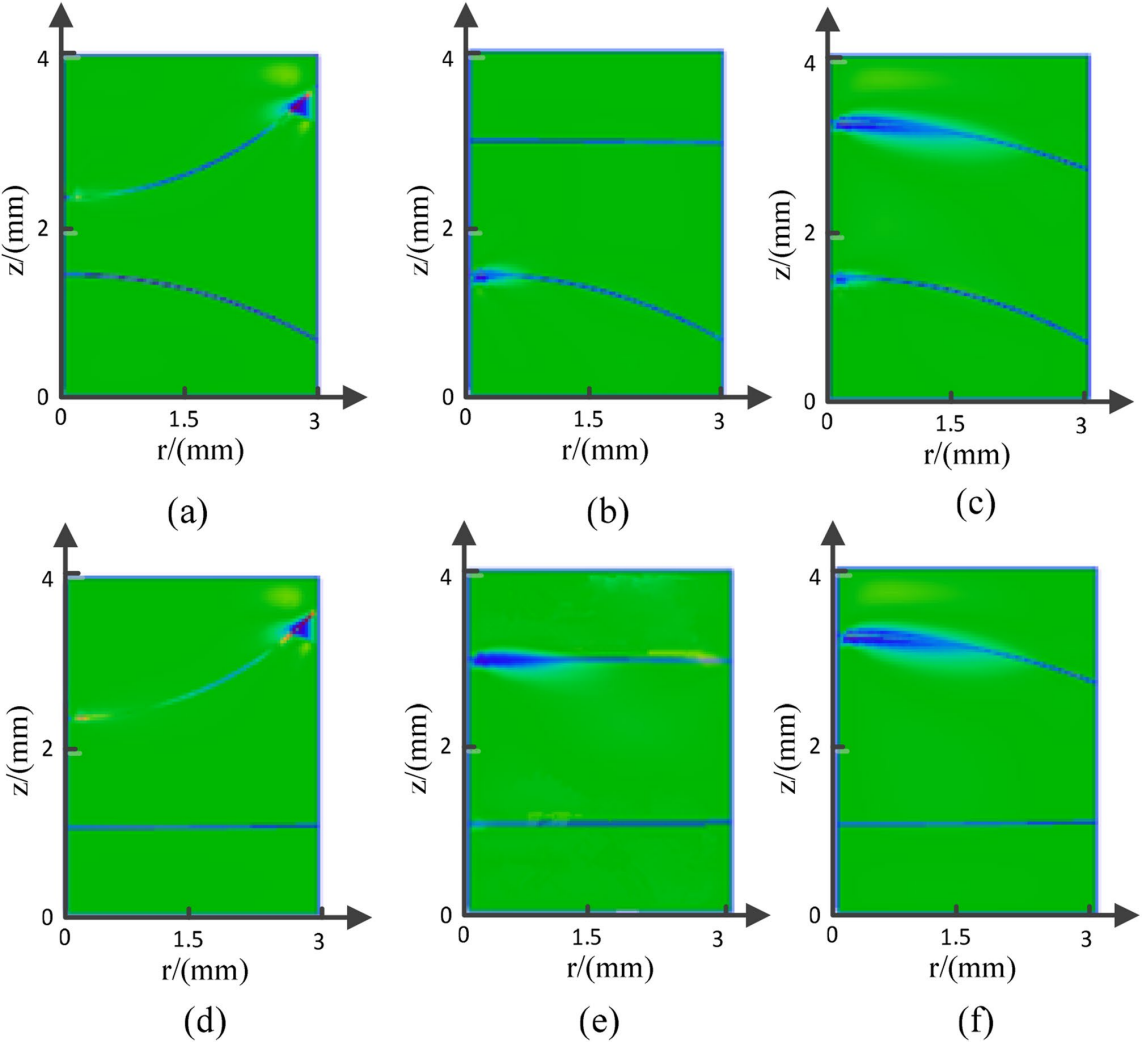
Meniscus of the EWOD lens with 6 mm optical aperture in equilibrium state: (**a**) *U*_*1*_ = 60 V, *U*_*2*_ = 20 V, (**b**) *U*_*1*_ = 60 V, *U*_*2*_ = 100 V, (**c**) *U*_*1*_ = 60 V, *U*_*2*_ = 120 V, (**d**) *U*_*1*_ = 100 V, *U*_*2*_ = 20 V, (**e**) *U*_*1*_ = 100 V, *U*_*2*_ = 100 V, (**f**) *U*_*1*_ = 100 V, *U*_*2*_ = 120 V.

We established the lens configuration in COMSOL Multiphysics, where the meniscus changes with the applied voltage, as shown in Fig. 9.

**Figure 10:**
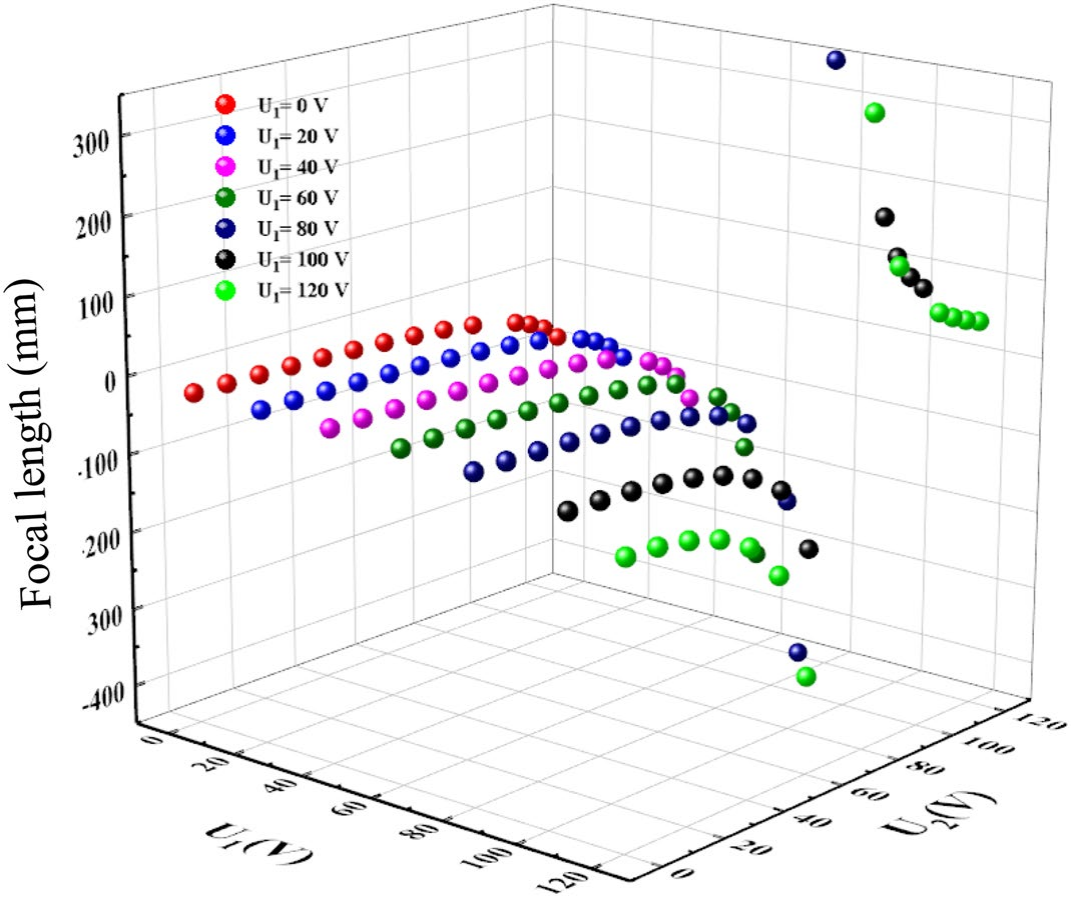
Focal length of the lens under different applied electric fields.

The focal length is expressed as

8 $$f = \frac{{n_{w} }}{{\frac{1}{{f_{1} }} + \frac{1}{{f_{2} }} - \left( {\frac{d}{{n_{g} }}} \right)\frac{1}{{f_{1} f_{2} }}}}$$

where $f_{1} ,f_{2}$ are the focal lengths of the two lenses that formed the electrowetting-based triple-liquid double zoom lens, and $d$ denotes the distance between the two lenses. The two lenses have the same focal length dynamic range and $d$ depends on $h\left( 0 \right)$, which changes with the focal length. When $f_{1} = - 35.394$ mm, $f_{2} = - 35.394$ mm, namely *V*_*1*_ = 0 V and *V*_*2*_ = 0 V, the maximum negative focal length *f* is − 22.87 mm. In contrast, when $f_{1} = 74.93$ mm, $f_{2} = 74.93$ mm, namely *V*_*1*_ = 120 V, *V*_*2*_ = 120 V, the minimum positive local length is 49.27 mm, as shown in Fig. 10.

**Figure 11:**
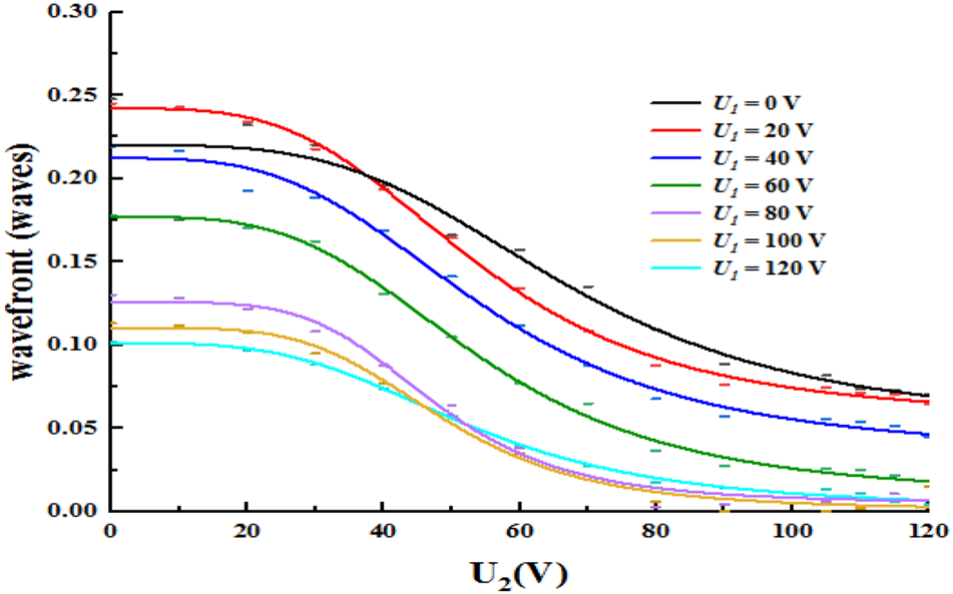
Dependence of wavefront aberration on the variable voltage (U_1_ and U_2_).

For a thick biological tissue, the light sheet fluorescence microscopy (LSMF) with a large axial scanning range and a high axial scanning accuracy is necessary. In LSMF, the axial scanning range depends on the focusing tunability range of the liquid lens. As shown in Fig. 10, the lens has a larger focal length dynamic range, which is (− ∞,− 22.87 mm) ∪ (49.27 mm, + ∞), and a higher accuracy for the focal length.The wavefront aberration is shown in Fig. 11.

As shown in Fig. 11, the wavefront aberration of the electrowetting-based triple-liquid dual-zoom lens depends on the variable voltage, including U_1_ and U_2_, which decreases slightly as the applied voltage increases, and is less than 1/4 waves in the zoom process.

## Discussion

The electrowetting lens has variable focal length characteristics resulting from the tunability of the liquid–liquid surface under the applied electric field. The liquid–liquid surface depends on the contact angle and optical aperture. Because of the effect of edge pinning, the contact angle is smaller than the theoretical value; the radius of the liquid–liquid surface does not have a strictly linear relation with the contact angle.

**Figure 12:**
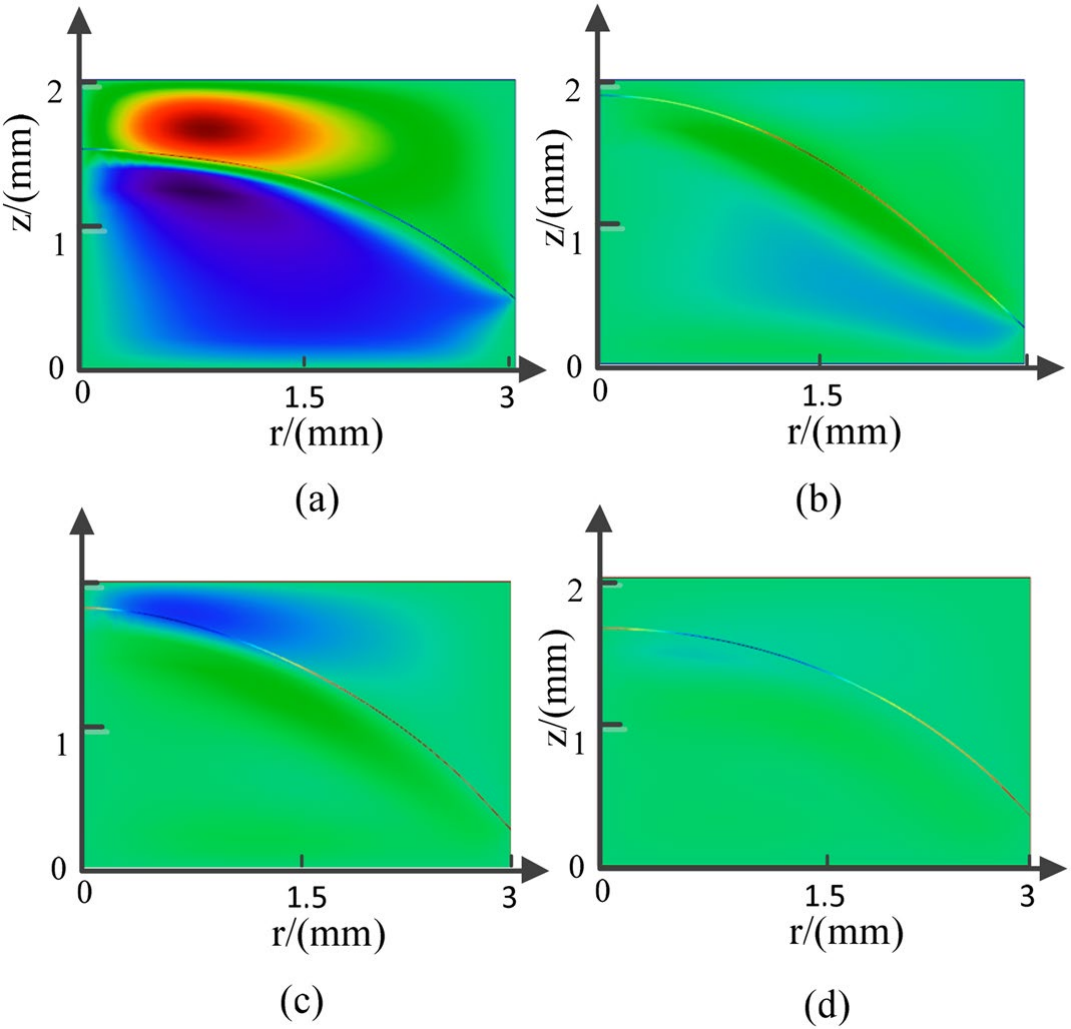
Dynamic of liquid–liquid surface (because of its symmetry, the figures show its section from the center to the tube edge) (**a**) t = 0.01 s, (**b**) t = 0.02 s, (**c**) t = 0.03 s, (**d**) t = 0.045 s.

The focusing property and wavefront aberration are the main properties of an electrowetting lens, the wetting effect changes under the applied electric field, and the forces are imbalanced. Considering a liquid rising up in a vertical capillary tube, there are five main forces incurred on the liquid: surface tension on the free surface, weight of the liquid column, viscosity force on the inner surface of the tube, inertia force, and applied force. The mean height of the liquid changes with the time before balancing the five forces, which reduces with the dynamic contact angle. Figure 12a–d show the dynamic liquid–liquid surface at V = 20 V. The optical aperture of the lens is 6 mm. At t = 0.045 s, the five forces are in equilibrium and the liquid–liquid surface is stable.

The focal length of electrowetting lens is related to the optical aperture, and thus, the numerical aperture decreases as the optical aperture increases. To expand the numerical aperture, we propose the electrowetting-based triple-liquid lens, which has a larger focal length range than the electrowetting-based double-liquid lens, and therefore, a larger numerical aperture.

## Conclusion

The liquid lens, as the main optical device, can realize the scanning imaging of the biological tissue. To promote its application in the LSFM system, the proposed lens should have a large numerical aperture and low wavefront aberration, which depend on the clear aperture, refractive index, and surface tension of the liquids. Thus, studying the relations among numerical aperture, wavefront aberration, is important. In the lens systems, the focal length depends on the refractive index and surface tension of the liquids. Therefore, we employ the focal length to characterize the refractive index and surface tension of liquids.

The focal length of the liquid lens can be tuned by changing the shape of the meniscus under the applied electric field. Because the meniscus is restructured as spherical waves, the reciprocal of the radius of meniscus and the square of the applied voltage, as well as the radius of meniscus and the optical aperture, are linearly related; the focal length is directly proportional to the radius of meniscus; and the wavefront aberration is inversely proportional to the optical aperture but directly proportional to the applied voltage. Numerical aperture is a main parameter for a lens, and depends on the focal length and optical aperture. It is directly proportional to the focusing property and inversely proportional to the optical aperture. The proposed electrowetting-based triple-liquid lens has a larger focal length range than a traditional electrowetting lens. For instance, focal length of the electrowetting lens with an liquid-liquid surface ranges as (− ∞,− 35.594 mm) U(74.930 mm, + ∞), while the proposed lens’ focal length ranges as (− ∞,− 22.870 mm) U(49.270 mm, + ∞). Moreover, the lens has low wavefront aberration. Because of the tunability of the liquid–liquid surface, we can precisely obtain the focal length. However, the thickness of the lens is larger than that of the double-liquid lens.

## Data Availability

All data generated or analyzed during this study are included in this published article.
